# Correlation of spontaneous radiographic cranial tibial translation with complete cranial cruciate ligament rupture and medial meniscal tears in dogs

**DOI:** 10.1371/journal.pone.0296252

**Published:** 2023-12-22

**Authors:** Nicholas J. Olson, F. Robert Weeren, Eric van Eerde

**Affiliations:** 1 Department of Surgery, BluePearl Pet Hospital, Tampa, Florida, United States of America; 2 Department of Radiology BluePearl Pet Hospital, Tampa, Florida, United States of America; Sheikh Hasina National Institute of Burn & Plastic Surgery, BANGLADESH

## Abstract

The primary objective of our study was to determine the prevalence of cranial tibial translation on a single unstressed, standing angle, mediolateral radiograph of the stifle and the accuracy of diagnosing complete cranial cruciate ligament rupture in dogs with this finding using a previously published method. The secondary objective was to determine if there was a higher incidence of meniscal injuries associated with spontaneous radiographic cranial tibial translation as previously proposed. Medical records were reviewed for client owned dogs with cranial cruciate ligament rupture that underwent surgical stabilization with intra-operative evaluation of the stifle joint via arthrotomy between June 2013 to January 2022 and had pre-operative radiographs performed within 60 days prior to surgery. Pre-operative radiographs were evaluated for cranial tibial translation via the previously published method. Three hundred twenty-three dogs met the inclusion criteria for the study. Intra-operative findings and radiographic assessments were evaluated for correlations. Cranial tibial translation on pre-operative standing angle radiographs detected cranial cruciate ligament tears in 24.8% of cases but had a positive predictive value of 97.5% for diagnosing complete cranial cruciate ligament rupture with a specificity of 95.4% and an overall accuracy of 36.8%. Meniscal tears were present in 58.75% of cases with radiographic cranial tibial translation and 41.25% of cases without. There was no significant increase in the incidence of meniscal tears between the two groups. The presence of radiographic cranial tibial translation in dogs on an unstressed, standing angle, mediolateral radiograph of the stifle is diagnostic for cranial cruciate ligament rupture, but cannot be used to determine the presence of a meniscal tear.

## Introduction

Cranial cruciate ligament (CCL) rupture is one of the most common orthopedic diseases seen in dogs. Prevalence of CCL rupture in the dog has been increasing significantly, previously reported to have more than doubled in a 40-year time span with an estimated $1.32 billion spent on treatment in one year alone [1,2]. Diagnosis of a CCL rupture is typically made with corroborating patient signalment, history, and physical exam findings consisting of hindlimb lameness, stifle instability in the cranial-caudal plane, stifle joint effusion with thickening of stifle, and radiographic evidence of joint effusion and osteoarthritis [3]. Commonly, the cranial drawer test (CDT) and tibial compression test (TCT) are utilized to evaluate for stifle laxity in the cranial/caudal plane where cranial displacement of the tibia during these tests are indicative of a CCL rupture. However, false negatives from the CDT can occur with larger patients or patients with high muscle tone, potentially necessitating sedation, or anesthesia for more accurate evaluation. While the TCT can provide a mechanical advantage for these patients by utilizing the gastrocnemius-calcaneal tendon mechanism to overcome higher muscle tone, a positive TCT is not detected as consistently as the CDT for many examiners [4]. While sedated orthopedic examination with these tests is routinely used to diagnose CCL rupture in specialty practice, at times, non-sedated radiographs are often the sole diagnostic obtained in general practice in patients with lameness. Establishing a method to identify spontaneous cranial tibial translation (CTT) on standard radiography and its correlation between complete CCL rupture and/or meniscal tears may allow for more prompt referral to a surgical center.

Radiographs of the stifle can aid in the diagnosis of a CCL rupture as well as evaluate for concurrent soft tissue or bony abnormalities. The “Cazieux-positive” sign, subjective cranial displacement of the proximal tibia in relation to the femur or spontaneous CTT, has previously been determined to always indicate a CCL rupture but is considered a rare finding with no recent reports evaluating true prevalence [5–7]. Other radiographic tests or measurements have been evaluated to diagnose CCL rupture such as tibial compression radiography, but require serial radiographs that include stressed views or special positioning to perform [5,8–10].

Meniscal tears are commonly seen in conjunction with CCL ruptures and are a significant cause of pain, inflammation, and lameness [11–13]. In dogs, incidence of meniscal tears are reported between 20–77% with numerous reports between 40–60% [11–17]. Physical exam findings such palpation of a “click” during range of motion of the stifle, pain on flexion of the stifle, and use of the modified TCT have been evaluated to diagnose meniscal tears with positive predictive values ranging from 67% to 84% [18–20]. One challenge with using physical examination to detect meniscal tears is that only certain types may be identified [21].

The primary objective of our study was to determine the prevalence of CTT on a single unstressed, standing angle, mediolateral radiograph in dogs and to see if a correlation to complete CCL rupture exists using the previously published method [22]. A second objective of our study was to determine if the presence of CTT on these radiographs was also correlated with meniscal tears as previously stated [22]. We hypothesized that the instance of CTT on an unstressed, standing angle, mediolateral radiograph will be low (less than 10%), but will correlate with complete CCL tears. We also hypothesized that dogs with spontaneous radiographic CTT will not have a significantly higher instance of meniscal injuries (25% increase in overall incidence) than dogs without spontaneous radiographic CTT.

## Materials & methods

### Study design and inclusion criteria

Medical records for this retrospective, multicenter cohort study from four regional specialty referral hospitals were reviewed for dogs receiving a tibial plateau leveling osteotomy (TPLO) or lateral suture stabilization as treatment for CCL rupture between January 2013 and January 2022. Dogs were included in our study if operative reports were available that confirmed CCL rupture and the status of the medial meniscus via stifle arthrotomy and there was a pre-operative radiograph of a standing angle, mediolateral projection of the affected stifle performed within 60 days prior to the date of surgery with 0 days representing radiographs being performed the day of surgery. Calibration of the radiographs were not necessary for inclusion in the study as none of the measurements required knowledge of the length of the lines created. Standing angle of the stifle was defined to be between 125° and 145°, encompassing a range of previously reported normal standing angles in various dog breeds [23]. Radiographs meeting these criteria with less than 75% overlap of the femoral condyles were excluded to allow for accurate measurement. Dogs with incomplete medical records, radiographs that did not meet the inclusion criteria, previous surgeries on the femur, stifle, or tibia or additional other abnormalities to the stifle other than concurrent medial patellar luxation (MPL) of any grade were excluded. Dogs with concurrent MPL were included as the incidence of concurrent MPL with CCL rupture in small dogs is reported to be between 22–41% [24]. Although MPLs are only reported to be present in 15% of large dogs, 41% of those dogs have been reported to have a concurrent CCL rupture [24].

### Radiograph interpretation

Radiographs were evaluated by two investigators (N.J.O. and F.R.W.) separately and blinded to CCL and meniscal status for the patients to limit bias. CTT was evaluated as pictured in Fig 18–11 in *Brinker*, *Piermattei*, *and Flo’s Handbook of Small Animal Orthopedics and Fracture Repair* [22]. Cases where there was disagreement in radiographic measurements between the investigators were submitted to the third investigator (E.V.), a board-certified veterinary radiologist, for final evaluation. Radiographs were evaluated on an online radiograph viewer (Asteris Keystone Omni).

**Fig 1:**
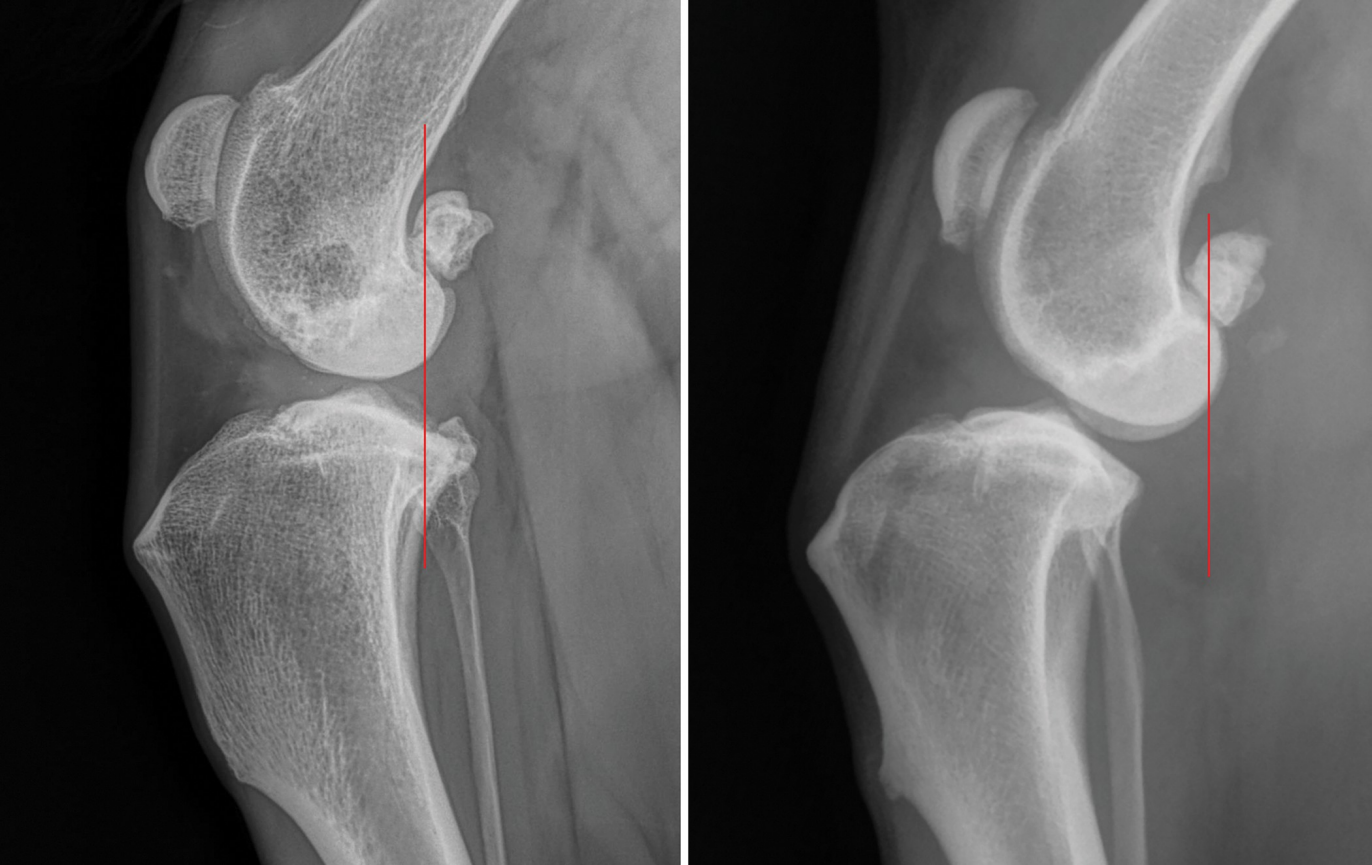
Examples of unstressed, standing angle, mediolateral radiographs of the stifle without (A) and with (B) cranial tibial translation.

The radiographs were rotated in the viewer program to closely replicate the appearance of the pictured template with the peaks of the trochlear ridges facing cranially to the left of the screen, the femoral condyles curving caudally to the right over the tibial plateau and the femoral diaphysis exiting towards the top right portion of the radiograph. A vertical line in relation to the computer monitor was placed through the caudal aspect of the femoral condyle to approximate its location on the template as pictured. The line was ensured to have passed through the fabella and extended distally to the crus. The location of the line through the fabella had mild variation due to individual anatomic variation and arthritic changes present. The stifle was determined to have CTT if the vertical line did not contact the tibial condyles or head of the fibula also as described in the referenced textbook (Fig 1). Measurement of the stifle angle was performed by measuring the angle of intersecting lines representing the anatomic axis of the femur and tibia. The anatomic axis was approximated in cases of narrowly collimated radiographs. Femoral condyle overlap was subjectively assessed, via estimation of overlap with no on-screen measurements.

### Statistical analysis

The response variables were presence of CTT in pre-operative radiographs and presence of a meniscal tear diagnosed during stifle arthrotomy during CCL stabilization surgery. The factors that were tested for any association with those response variables were age, sex, weight, presence of a concurrent MPL, the length of time between performing the radiographs and performing surgery, partial or complete CCL tears, and either the presence of a meniscal tear diagnosed during surgery or presence of CTT in pre-operative radiographs respectively for the alternate response variable. Many data sets were not normally distributed (Shapiro Wilk test of normality). Univariate statistics of all comparisons was performed, by either Wilcoxon rank sum test or chi-square/Fisher’s exact test, as appropriate. Descriptive statistics (mean, standard deviation, standard error of the mean, median, min/max, and 25th/75th quartiles) were reported.

Multivariate statistics (multiple logistic regression) with the above response variables (yes/no) and factors was completed. Multicollinearity was assessed by means of the variance inflation factor, where < 2.5 was acceptable. Linearity of the log odds versus the continuous factors was assessed by means of the Box Tidwell approach (inclusion of a quadratic term). If the relationship was nonlinear, then the quadratic term was included in the model. All factors were initially included in the model. Factors with the highest p value were excluded one at a time, retaining all factors with p < 0.10. P values, odds ratios, and 95% confidence limits were reported. In addition, probabilities and risk ratios were calculated from the logistic model. If a quadratic term was included, plots were generated of the relationship. All calculations were by means of statistical software (NCSS statistical software).

## Results

During the studied period, 323 cases met the inclusion criteria. Sixty-two breeds were identified with only 12 breeds represented five or more times (Table 1). The remaining overall patient data and identified stifle abnormalities can be seen in Table 2. The data was analyzed for correlations with spontaneous CTT on pre-operative radiographs (Table 3) and meniscal tears (Table 4). There were 3 cases with grade I MPL, 12 cases with grade II MPL, 20 cases with grade III MPL, 3 cases with grade IV MPL, and 5 cases without an MPL grade recorded.

**Table 1:** Breeds composition of the patient population with the number of cases for each breed.

Breed	Number
Mixed breed dog	124
Labrador Retriever	20
Pitbull, Yorkshire Terrier	16
Bichon Frise	12
Boxer, English Bulldog, German Shepherd, Golden Retriever	8
Rottweiler	7
Doberman Pinscher, Havanese	5
American Bulldog, Australian Shepherd, Catahoula Leopard Dog, Maltese, Miniature Poodle	4
Black Mouth Cur, Border Collie, Puggle, West Highland White Terrier	3
Australian Blue Heeler, Beagle, Cavalier King Charles Spaniel, Corgi, German Shorthaired Pointer, Jack Russell Terrier, Mastiff, Miniature Australian Shepherd, Retriever, Scottish Terrier, Shih Tzu, Terrier, Toy Poodle	2
Akita, Australian Cattle Dog, Bernese Mountain Dog, Brittany Spaniel, Bulldog, Cairn Terrier, Chihuahua, Cocker Spaniel, English Setter, Great Dane, Husky, Irish Wolfhound, Labradoodle, Lhasa Apso, Miniature Pinscher, Newfoundland, Nova Scotia Duck Retriever, Petit Basset Griffon Vendeen, Pointer, Pomeranian, Poodle, Portuguese Water Dog, Schipperke, Schnoodle, Siberian Husky, Standard Schnauzer, Weimaraner, Whippet	1

**Table 2:** Demographics and stifle abnormalities present in the entire studied population.

Factor	Number
Complete CCL rupture–# of cases/(% of cases)	280 (86.69%)
Partial CCL rupture–# of cases/(% of cases)	43 (13.31%)
Concurrent MPL–# of cases/(% of cases)	43 (13.31%)
Meniscal tears–# of cases/(% of cases)	162 (50.15%)
Radiographic CTT–# of cases/(% of cases)	80 (24.77%)
Spayed females–# of cases/(% of cases)	183 (56.66%)
Intact females–# of cases/(% of cases)	4 (1.24%)
Neutered males–# of cases/(% of cases)	132 (40.87%)
Intact males–# of cases/(% of cases)	4 (1.24%)
Weight–median/(IQR) in kg	26.00 (9.60–34.30)
Age–median/(IQR) in years	7.51 (4.80–9.42)

**Table 3:** Compared demographics and stifle abnormalities present between groups without and with CTT on pre-operative radiographs.

Factor	Without CTT on pre-operative radiographs	With CTT on pre-operative radiographs
Total cases–# of cases/(% of cases)	243 (75.23%)	80 (24.77%)
*Complete CCL rupture–# of cases/(% of cases)	202 (83.13%)	78 (97.5%)
Partial CCL rupture–# of cases/(% of cases)	41 (16.87%)	2 (2.5%)
Concurrent MPL–# of cases/(% of cases)	28 (11.52%)	15 (18.75%)
Meniscal tears–# of cases/(% of cases)	115 (47.32%)	47 (58.75%)
Females–# of cases/(% of cases)	146 (60.08%)	41 (51.25%)
Males–# of cases/(% of cases)	97 (39.92%)	39 (48.75%)
*Weight–median/(IQR) in kg	28.7 (10.90–35.50)	12.90 (8.33–27.25)
*Age–median/(IQR) in years	7.25 (4.54–9.04)	8.76 (6.01–10.85)
Stifle angle–median/(IQR) in °	134.30 (130.50–139.10)	135.05 (131.00–138.38)
Time between radiographs and surgery–median/(IQR) in days	2 (0–13)	0 (0–11)

* p < 0.05.

**Table 4:** Compared demographics and stifle abnormalities present between groups without and with meniscal tears.

Factor	Without meniscal tears	With meniscal tears
Total cases–# of cases/(% of cases)	161 (49.85%)	162 (50.15%)
*Complete CCL rupture–# of cases/(% of cases)	125 (77.64%)	155 (95.68%)
Partial CCL rupture–# of cases/(% of cases)	36 (22.36%)	7 (4.32%)
*Concurrent MPL–# of cases/(% of cases)	30 (18.63%)	13 (8.02%)
*Females–# of cases/(% of cases)	84 (52.17%)	103 (63.58%)
Males–# of cases/(% of cases)	77 (47.83%)	59 (36.42%)
*Weight–median/(IQR) in kg	25.30 (8.50–33.85)	26.35 (10.95–35.10)
*Age–median/(IQR) in years	7.19 (4.04–9.32)	7.84 (5.03–9.44)
Stifle angle–median/(IQR) in °	135.00 (130.75–140.20)	133.90 (130.50–138.33)
Time between radiographs and surgery–median/(IQR) in days	1 (0–13)	2 (0–13)

* p < 0.05.

No significant association was identified between the presence of CTT on pre-operative radiographs and the presence of concurrent meniscal tears. Significant associations identified with the presence of CTT on pre-operative radiographs were complete CCL rupture and patients of older ages and lower weights. Significant associations identified with meniscal tears were complete CCL rupture, the absence of a concurrent MPL and female dogs. Using CTT on pre-operative radiographs as a diagnostic test for complete CCL tears yielded a sensitivity of 27.9%, specificity of 95.4%, positive predictive value of 97.5%, and negative predictive value of 16.9% with an overall accuracy of 36.8%.

## Discussion

The presence of CTT on an unstressed, standing angle, mediolateral radiograph of the stifle was previously reported as a rare occurrence and secondary to a torn meniscus that has caused a wedge preventing the stifle from returning to its normal anatomic position [22]. To the authors’ knowledge a true prevalence has not been reported. In our study, spontaneous radiographic CTT was present in nearly 25% of cases. Complete CCL rupture has been shown to have a relatively higher degree of instability versus partial CCL rupture [5,8,25]. Our study population included a higher number of dogs with complete CCL rupture than partial CCL rupture with only two of the dogs with partial CCL rupture exhibiting spontaneous radiographic CTT. Discordant numbers in the two populations may have skewed the prevalence of spontaneous radiographic CTT for dogs with CCL rupture overall. When evaluating dogs with only complete or partial CCL rupture, the prevalence of spontaneous radiographic CTT was 27.9% and 4.7% respectively, thereby correlating complete CCL rupture with spontaneous radiographic CTT.

The mechanism for spontaneous CTT is unknown. Previously, spontaneous radiographic CTT was proposed to be secondary to a meniscal tear creating a wedge and preventing the tibia from returning to its normal resting place from CTT [22]. However, that theory, while still a potential mechanism for spontaneous radiographic CTT, was not supported in our population as over 40% of dogs with spontaneous radiographic CTT had intact menisci. Previous studies evaluating for a distance change in serial radiographs for CTT also have not found a correlation with meniscal status. These studies have had low numbers of cases with meniscal tears (less than 20 total) [5,25]. Our population contained 162 cases with meniscal tears providing stronger support for a lack of correlation and reducing the potential for a type II error.

Given the complexity of the stifle joint, we suspect the causes for spontaneous CTT to be multifactorial and may include chronicity of injury, muscle mass, muscle tone, and TPA among others. With CCL rupture, CTT typically occurs secondary to weightbearing forces and can be induced during physical examination with the TCT. Yet, there is evidence that CTT occurs during all phases of the gait cycle in CCL deficient stifles [26]. Therefore, weightbearing forces may not be required to induce CTT or the removal of weightbearing forces may not completely resolve CTT leading to spontaneous or persistent CTT. Utilizing the methods from our study, a single radiograph can provide a result that is highly predictive (97.5%) and specific (95.6%) for a complete CCL rupture and provides an additional method for diagnosing complete CCL tears. Although, lack of CTT does not imply an intact CCL as CCT was only present in approximately 25% of complete CCL rupture cases. Partial CCL rupture also cannot be completely ruled out as two cases with partial CCL rupture were also positive for spontaneous radiographic CTT in this population. Further details regarding the degree of rupture or competency of the remaining ligament in these cases were not available. As only two cases of partial CCL rupture exhibited CTT, it is possible that these cases were more advanced partial tears with the remaining intact ligament being incompetent acting as a functionally complete CCL tear. Other possibilities include misidentification of a complete CCL tear in surgery or error in reporting the CCL status in the operative report.

The technique used in this study to identify CTT has not been evaluated in any previous studies to the authors’ knowledge, although it has been published with the claim of identifying CTT on a standing angle, mediolateral radiograph of the stifle [22]. In our study, this technique was highly predictive and specific for complete CCL rupture, although the sensitivity and accuracy were low. Positive predictive values can be altered based on prevalence of a disease. In our study, the prevalence for complete CCL tears was high (86.7%) as the cases used in the study were identified because they had surgery for a CCL tear. The overall prevalence of CCL tears among all dogs has been reported to be between 3–5% which would lower the positive predictive value of this technique [2,27]. However, the specificity is unchanged by prevalence of the disease and the technique remains highly specific for complete CCL tear when identified. Additionally, this technique would likely be utilized for patients that are suspected to have CCL tears, a population likely to have a higher prevalence of CCL tears as opposed to the overall patient population. As radiographic CTT was only present in approximately 25% of cases, if CTT is not identified on radiographs using this technique, further investigation should be considered if CCL rupture is still suspected. If CTT is not present in the radiograph, the practitioner can consider maximally flexing the tarsus during a following radiograph and measure for CTT via other described techniques for stress radiography in the standing angle [8,10]. While the measurement method in our study has not been evaluated during stress radiography and requires further study to confirm its use, the authors suspect that utilizing the evaluated measurement in our study would aid in confirmation of a “Cazieux positive” sign but with unknown success.

Our data identified two factors, weight and age, to be associated with spontaneous CTT on radiographs with older dogs or dogs that weighed less to be more likely to have spontaneous CTT on radiographs. Previous studies have evaluated factors such as age and weight for a correlation to CCL rupture or contralateral CCL rupture with higher weight being identified in some studies and not in others [28–32]. Previous studies evaluating for CTT on serial radiographs have not evaluated risk factors for CTT [5,8,9,33,34]. We theorize that our findings may be attributable to these patients having less muscle mass or muscle tone to stabilize the stifle and maintain these patients in the normal anatomic position. Additional studies to evaluate this theory are needed. While not evaluated in this study, a steeper tibial plateau angle (TPA) could have been a contributing factor for CTT. Steeper TPAs have been reported in small dogs when compared to large dogs [35]. The role of TPA in CCL rupture has been debated. Some reports have shown no effect from TPA regarding the presence or progression of CCL rupture while others have identified earlier onset lameness and CCL rupture for steeper TPAs [35]. To the author’s knowledge, a correlation between TPA and identifying CTT has not been evaluated and further studies are needed to determine any potential link as this was outside the scope of our study.

Meniscal tears were not correlated with spontaneous radiographic CTT in our study. However, male dogs and dogs with a concurrent MPL were less likely to have meniscal tears in our study. To the authors’ knowledge, no sex predilection has been previously reported regarding meniscal tears and previous studies have not found a correlation between sex and CCL tears [28–32]. An increased incidence of meniscal tears for dogs with CCL rupture and concurrent MPL has not been reported. Similarly, our findings of male dogs being less likely to have meniscal tears, we theorize that these patients may be more affected by their lameness and receiving surgical repair earlier, thus avoiding chronic CCL rupture and subsequent increased risk of meniscal tears. Male dogs have been shown to have a significant difference in their medial and lateral TPAs with the lateral TPA being steeper than the medial TPA, while a significant difference was not identified in female dogs [36]. In humans, the ratio of medial TPA and lateral TPA is correlated to peak tibial rotation angle with an increased lateral TPA increasing the peak knee internal rotation and abduction angles [36]. Therefore, male dogs may be predisposed to additional forces on the CCL from internal rotation leading to further instability when CCL rupture has occurred and potentially a more significant lameness from the increased instability. This may lead to earlier surgical intervention which may reduce the instance of meniscal injury as meniscal injuries are correlated with chronicity of CCL rupture [37]. However, this finding has not been appreciated clinically and additional studies are needed to further evaluate this conclusion [28].

Limitations of our study include those inherent of a retrospective study. While attempts to standardize the radiographs evaluated were taken, some variation remained present as radiographs were performed by multiple people at multiple hospitals and without use of a positioning device. Complete knowledge of exact patient positioning cannot be known and could have increased the number of patients with CTT if stresses were being applied to the limb. Additionally, information regarding whether sedation or anesthesia was used to facilitate the radiographs or whether the patient was recently palpated for instability prior to obtaining the radiographs was not available, and their impact is unknown as muscle mass and tone may be a contributing factor to spontaneous CTT. The measurement technique utilized in this study had limitations in its application. The written description of the technique was vague in outlining an exact location for the vertical line to be placed, the relation of the vertical line to any features or structures, and any feature or structure to use to place the image in the appropriate orientation for measurement. Due to limited written description, utilizing this technique was mostly performed by attempting to recreate the appearance of the template image. A wide range of documented standing stifle angles were included in our study to encompass normal angles for most breeds which may have included some dogs within the allowed range, but outside of their normal standing stifle angle [23]. Additionally, anatomic differences between breeds, TPA, osteoarthritic changes, and mild anatomic abnormalities were not controlled for in our study. The impact of these variables is unknown and outside the scope of our study. All evaluations of the meniscus were performed via arthrotomy as opposed to arthroscopy. Meniscal evaluation through arthrotomy has been shown to be less sensitive and less specific than arthroscopic evaluation and may have led to misdiagnosis of the meniscal status in some of the cases evaluated [16]. From a clinical perspective, arthrotomy remains a common and accepted option for assessing meniscal status during CCL stabilization surgeries which may cause the data to parallel what veterinary surgeons are seeing clinically. The measurement method was not evaluated against normal stifles in our study. Partial CCL rupture has been shown to have an increased distance in bony structures or landmarks when evaluating for radiographic CTT via tibial compression radiography in previous studies when compared to normal stifles [8,25]. Given this finding and that only two of the 43 cases with partial CCL rupture were positive for spontaneous radiographic CTT, we conclude that this measurement technique always indicates a CCL rupture and would exclude normal stifles. However, additional studies applying the measurement method to a normal control group would be needed.

## Conclusions

The prevalence of CTT on an unstressed, standing angle, mediolateral radiograph of the stifle in our study surpassed our estimation. Our study supported our hypotheses that spontaneous radiographic CTT using this measurement method correlates with complete CCL rupture and is not correlated with increased incidence of concurrent medial meniscal tears. Radiographs with spontaneous CTT measured with this method cannot be used to determine the presence of meniscal injuries in dogs and is highly predictive for complete CCL rupture. Absence of radiographic CTT utilizing this method does not rule out CCL rupture as radiographic CTT was only present in approximately 25% of cases. Alternative methods are recommended to evaluate for the presence of concurrent meniscal injuries or partial CCL rupture.

## Supporting information

S1 File Raw data with analysis results. (XLSX)
